# Patterns and Determinants of Ecological Uniqueness in Plant Communities on the Qinghai-Tibetan Plateau

**DOI:** 10.3390/plants14152379

**Published:** 2025-08-01

**Authors:** Liangtao Li, Gheyur Gheyret

**Affiliations:** 1 College of Geographical Sciences and Tourism, Xinjiang Normal University, Urumqi 830054, China; llt2210383762@163.com; 2 Xinjiang Key Laboratory of Lake Environment and Resources in Arid Zone, Urumqi 830054, China

**Keywords:** climate, community characteristics, β-diversity, rare species, soil

## Abstract

The Qinghai-Tibetan Plateau is one of the world’s most prominent biodiversity hotspots. Understanding the spatial patterns of ecological uniqueness in its plant communities is essential for uncovering the mechanisms of community assembly and informing effective conservation strategies. In this study, we analyzed data from 758 plots across 338 sites on the Qinghai-Tibetan Plateau. For each plot, the vegetation type was classified, and all plant species present, along with their respective abundance or coverage, were recorded in the database. To assess overall compositional variation, community β-diversity was quantified, while a plot-level approach was applied to determine the influence of local environmental conditions and community characteristics on ecological uniqueness. We used stepwise multiple regressions, variation partitioning, and structural equation modeling to identify the key drivers of spatial variation in ecological uniqueness. Our results show that (1) local contributions to β-diversity (LCBD) exhibit significant geographic variation—increasing with longitude, decreasing with latitude, and showing a unimodal trend along the elevational gradient; (2) shrubs and trees contribute more to β-diversity than herbaceous species, and LCBD is strongly linked to the proportion of rare species; and (3) community characteristics, including species richness and vegetation coverage, are the main direct drivers of ecological uniqueness, explaining 36.9% of the variance, whereas climate and soil properties exert indirect effects through their interactions. Structural equation modeling further reveals a coordinated influence of soil, climate, and community attributes on LCBD, primarily mediated through soil nutrient availability. These findings provide a theoretical basis for adaptive biodiversity management on the Qinghai-Tibetan Plateau and underscore the conservation value of regions with high ecological uniqueness.

## 1. Introduction

Understanding the spatial structure of species diversity and its underlying drivers has been a central focus in ecological research for decades [1,2,3,4]. Whittaker introduced a hierarchical framework divided into three components: α-diversity, β-diversity, and γ-diversity [5]. Among these, β-diversity, representing species turnover between habitats, is a key metric of community heterogeneity. It is essential for elucidating community assembly processes, biogeographic patterns, and species replacement across landscapes [6,7,8,9]. Recent studies have examined β-diversity through multiple lenses, including environmental constraining factors, habitat boundary definition, and mechanistic modeling. Xiao et al. (2025) [10] proposed a novel theoretical framework for predicting α- and β-diversity across spatial scales in multi-taxa communities (e.g., plants, animals, microbes). By applying this model to global forest inventories, they identified the proportion of low-abundance species (PL) as a key predictor, accounting for approximately 85% of the variance in plant β-diversity along latitudinal gradients [10]. He et al. (2024) developed a scale-explicit framework to assess β-diversity patterns and drivers across spatial scales spanning local to regional extents [11]. These efforts consistently reveal spatial gradient patterns across taxa and biogeographic regions [8,12]. Research on the limiting factors of β-diversity currently dominates the literature and carries greater ecological significance than studies focused on spatial scales. For example, Guo et al. (2018) demonstrated that, in tropical karst seasonal rainforests, β-diversity was jointly governed by deterministic and stochastic processes, with environmental filtering emerging as the dominant mechanism [13], and Hu et al. (2024) [14] investigated the relationship between β-diversity and environmental factors across three vertical layers (herbs, shrubs, and trees) in tropical karst seasonal rainforests. They found that, due to environmental filtering, only species adapted to specific environmental conditions can survive. Within these three vertical layers, environmental filtering led to substantial changes in species composition across different layers, thereby influencing β-diversity [14]. Environmental factors impose stronger constraints in semi-arid and arid regions under low precipitation regimes. Li et al. (2024) demonstrated that environmental factors (climate and soil) exerted significantly stronger effects on plant β-diversity and its turnover component than spatial distance in the Dunhuang North Mountain and Mazong Mountain regions of the Gobi Desert, with habitat filtering identified as the dominant mechanism [15]. In addition, Al-Mutairi (2017) demonstrated that environmental constraints (particularly soil physicochemical properties) significantly drive β-diversity patterns in Saudi Arabia’s Tabuk desert flora, with anthropogenic disturbances further exacerbating taxonomic distinctness degradation [16].

Legendre and De Cáceres introduced two indices—local contribution to β-diversity (LCBD) and species contribution to β-diversity (SCBD)—to quantify the ecological uniqueness of communities [17]. Ecological uniqueness refers to the degree to which a local plant community exhibits distinct species composition, functional traits, or ecological responses compared to other communities within a regional species pool. This uniqueness arises from environmental filtering (e.g., climate, soil), biotic interactions (e.g., competition), and dispersal limitations, leading to site-specific biodiversity patterns. Studies of ecological distinctiveness contribute to our understanding of how plant communities are constructed in a given region. A higher LCBD value indicates a more unique species composition, often driven by unique local environmental conditions or the presence of non-native species [18,19]. LCBD is widely used to identify ecologically heterogeneous regions and has been applied in research on community assembly, responses to environmental gradients, and conservation prioritization [20,21]. In contrast, SCBD quantifies the contribution of individual species to overall β-diversity, reflecting the extent of their spatial variation and ecological functions [22,23]. Previous studies have shown that community characteristics—such as species richness and community vegetation coverage—as well as species’ niche properties, significantly influence both LCBD and SCBD values [19,24,25]. Therefore, combining LCBD and SCBD can provide a comprehensive understanding of the multi-scale processes that shape community structure.

While community-level β-diversity decomposition (LCBD/SCBD) is increasingly applied in biodiversity research, current studies show a pronounced bias toward aquatic ecosystems—spanning stream insects [26], urban benthic fauna [27], floodplain fish [28], lake zooplankton [20], crater pond biodiversity [29], and freshwater diatoms [19]. In contrast, terrestrial applications, especially in forest communities, remain limited [30,31,32]. The Qinghai-Tibetan Plateau (‘Roof of the World’) provides an ideal system for studying plant community ecological uniqueness, hosting two global biodiversity hotspots [33] and ~50% of China’s vascular plant species [34]. Despite accelerating climate threats [35], its complex topography–climate–edaphic interactions sustain diverse habitats [36]—yet large-scale β-diversity analyses remain limited. This study aims to explore the structural characteristics of plant ecological uniqueness in the Qinghai-Tibet Plateau through research on plant diversity in the region, advancing the understanding of species coexistence, functional trait distribution, and the ecological resilience of plant communities, thus providing new insights into how unique species compositions emerge across environmental gradients. This contributes to existing theoretical frameworks in community assembly and biodiversity conservation on the Qinghai-Tibet Plateau. In addition, the findings related to the relationship between species richness and vegetation coverage can assist in the sustainable management of natural resources. For example, understanding how community coverage affects ecological uniqueness could inform land restoration projects or agriculture practices in the region, ensuring that biodiversity is maintained while minimizing human impact. For this purpose, we analyzed vegetation survey data from 758 plots across the Qinghai-Tibetan Plateau, applying the LCBD-SCBD indices to systematically assess the spatial distribution of ecological uniqueness in plant communities and identify key environmental drivers. The primary goals of this study are formulated as follows: (1) characterize the geographic patterns of ecological uniqueness in plant communities, (2) identify the environmental factors influencing the ecological uniqueness of plant communities on the Qinghai-Tibetan Plateau and evaluate their relative importance. To establish a scientific basis for regional biodiversity conservation and ecological management, this study’s results are projected to identify key species and regions demanding conservation priority. Based on existing theory and the unique environmental context of the Qinghai-Tibetan Plateau, we propose the following hypotheses: (1) Ecological uniqueness (LCBD) will exhibit significant spatial gradients along longitude, latitude, and elevation, driven by topographic heterogeneity and rare species distribution. (2) Woody species (shrubs and trees) will contribute more strongly to β-diversity than herbaceous species due to their niche specialization in extreme environments.

## 2. Materials and Methods

### 2.1. Study Areas and Plant Community Survey

**Figure 1:**
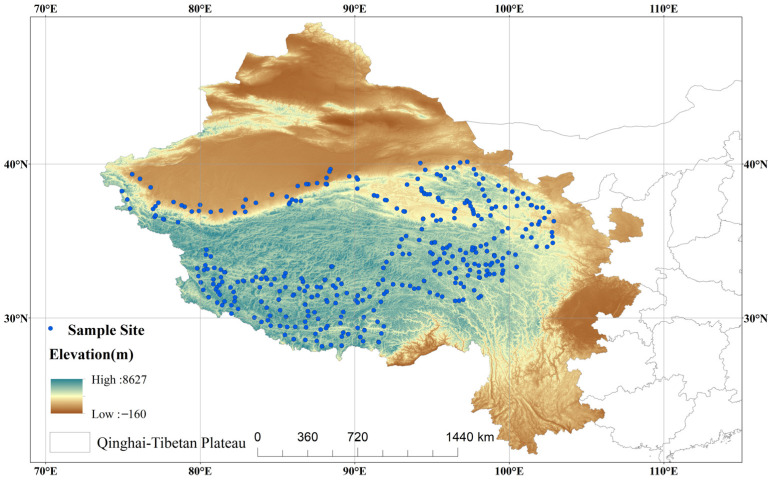
Distribution of sampling sites across the Qinghai-Tibetan Plateau.

This study focused on several representative regions of the Qinghai-Tibetan Plateau, including the Ali and Sanjiangyuan regions; the southern Tibetan mountains; the Qaidam Basin; and the high mountain, oasis, and desert areas of the Kunlun, Altun, and Qilian Mountains. The plant community data used in this study were obtained from the Qinghai-Tibetan Plateau vegetation plot Database (Jin et al., 2022, Chinese Journal of Plant Ecology, 46, 846–854. DOI: 10.17521/cjpe.2022.0174 [37]). The fieldwork was conducted during July and August from 2018 to 2021, focusing on vegetation composition and community structure. Data collection targeted alpine meadow, grasslands, shrublands, deserts, temperate meadows, and temperate deserts. At each site, 1 to 3 representative plots were randomly selected for investigation. In meadow and grassland communities, three 1 m × 1 m plots were established per site; in shrublands, two 2 m × 2 m plots; and in deserts, one to two plots of 5 m × 5 m or 10 m × 10 m were set up. Due to hazardous terrain conditions on the Tibetan Plateau (e.g., steep slopes or unstable ground), the size of some sampling plots was reduced from the standard protocol, with compensatory adjustments made to plot numbers where feasible, to ensure data representativeness and researcher safety. All plant species occurring in each sample plot were recorded. Collected data included species name, phenological stage, number of individuals or clumps, leaf layer height and reproductive branch height for herbaceous plants, canopy height for woody plants, and diameter at breast height (DBH) for trees or basal diameter for shrubs. Furthermore, plant community coverage (defined as the proportion of ground area covered by foliage) was estimated visually as the sum of horizontal projection areas of all above-ground plant organs within each sample plot. In addition to plot ID and geographic coordinates, environmental variables were recorded for each plot, including elevation, slope, aspect, disturbance status, and total vegetation coverage. For each sampling quadrat, geographic coordinates (longitude, latitude) and elevation were measured using GPS, aspect was determined with a compass (0° = true north, 180° = true south), slope angle was quantified using a clinometer, and anthropogenic disturbance status was obtained from local records. In total, the survey included 338 sites and 758 plots, comprising 107 alpine meadow plots, 87 alpine steppe plots, 38 alpine shrub plots, 16 alpine desert plots, 15 temperate meadow plots, 7 temperate steppe plots, 64 temperate desert plots, and 4 forest plots (Figure 1). In total, 837 species from 279 genera and 65 families were recorded, yielding 6551 species occurrence records. Plant nomenclature and taxonomic classification were based on the Flora of China (Editorial Committee, 1959–2004), and subsequently updated using the iPlant database (http://www.iplant.cn/, accessed on 5 January 2024). The growth form for each species was also obtained from this source. We categorized plant species into four growth forms—herbs, shrubs, trees, and lianas—to assess their respective contributions to β-diversity. Among these, herbaceous plants were the most abundant, comprising 699 species, followed by 123 species of shrubs, 8 species of lianas, and 7 species of trees. Due to insufficient sampling, lianas were excluded from subsequent analyses.

### 2.2. Environmental Variables

To characterize the physicochemical properties of soil on the Qinghai-Tibetan Plateau, we selected six key indicators: soil pH (pH), total nitrogen content (TSN), total phosphorus content (TSP), bulk density (BD), organic carbon content (OC), and soil moisture (SM). The first five indicators were obtained from the Chinese Soil Database published by Shangguan et al. (2013), while soil moisture data were sourced from the GLEAM v3.7 Datasets (www.gleam.eu, accessed on 20 November 2023). The Chinese Soil Database provides measurements across eight soil layers from 0 to 2.296 m in depth: 0–0.045 m, 0.045–0.091 m, 0.091–0.166 m, 0.166–0.289 m, 0.289–0.493 m, 0.493–0.829 m, 0.829–1.383 m, and 1.383–2.296 m. In this study, we focused on the top 0–0.289 m layer and calculated weighted mean value using data from the top four layers. The calculation is defined as:

(1) $$MS=\frac{s_{1}*d_{1}+s_{2}*d_{2}+s_{3}*d_{3}+s_{4}*d_{4}}{d_{1}+d_{2}+d_{3}+d_{4}}$$

where MS denotes the weighted mean value, s1~s4 represent the values of each layer, and d1~d4 correspond to their respective depths.

In order to quantify the impact of climatic factors on plant community diversity, we selected five key meteorological variables based on the current climatic conditions of the Qinghai-Tibet Plateau: mean annual precipitation (MAP), precipitation seasonality (PS), mean annual temperature (MAT), temperature seasonality (TS), and solar radiation (SRAD). We extracted these variables from the WorldClim database (http://www.worldclim.org/, accessed on 22 November 2023) using the geographic coordinates of each sampling point at a resolution of 1 km × 1 km.

### 2.3. Calculation of Beta Diversity and the Ecological Uniqueness of Plant Communities

Total community coverage was calculated by summing individual species coverages within each sample plot. Plot-level average coverage was then derived by dividing total coverage by species richness.

In this study, we quantified plant community beta diversity (β-diversity) using the method proposed by Legendre and De Cáceres [16], which defines β-diversity as the total variance of the species coverage matrix of the plant community (BD_total_). The calculation is as follows:

(2) $$S_{\mathrm{ij}}\;=\;(Y_{\mathrm{ij}}\;-\;{\bar{Y}}_{i})$$

(3) $${SS}_{total}=\sum_{i=1}^{n}\sum_{j=1}^{p}S_{ij}$$

BD_total_ = Var(Y) = SS(Y)/(n − 1) (4)

where Y_ij_ is the coverage of species *j* in plot *i*, $\bar{Y}$_i_ is the mean coverage values of species *j* across all plots, n is the number of plots, and S_ij_ is the squared deviation of the coverage of species *j* in plot *i* from the mean coverage of that species across all plots; SS_total_ represents the total sum of squares of the species coverage matrix, and BD_total_ is the unbiased estimator of variance, which can be used to quantify community β-diversity.

Legendre and De Cáceres [17] further partitioned this total variance into contributions from individual species and sampling units. This decomposition enables quantification of both species-level and community-level uniqueness within the β-diversity framework. The specific calculation is as follows:

(5) $${SS}_{j}\;=\;\sum_{i\;=\;1}^{n}S_{ij}$$

(6) $${SCBD}_{j}={SS}_{j}/{SS}_{total}$$

(7) $${SS}_{i}=\sum_{j=1}^{p}S_{ij}$$

(8) $${LCBD}_{i}={SS}_{i}/{SS}_{total}$$

where SS_j_ is the total sum of squares of coverage deviations for species *j*, while SS_i_ represents the total sum of squares of deviations for all species in sampling unit or community *i.* The species contribution to β-diversity (SCBD_j_) quantifies the extent to which species *j* contributes to overall community variability. In contrast, the local contribution to β-diversity (LCBD_i_) captures the compositional distinctiveness of plot i, thus reflecting its ecological uniqueness. A *t*-test was performed on the processed SCBD data for the different plant types to assess data validity.

### 2.4. Statistical Analysis

All environmental variables—latitude, longitude, elevation, and edaphic and climatic variables—were standardized before analysis. To eliminate unit differences and ensure comparability, each variable was scaled so that it had a mean of zero and a standard deviation of one. We examined the relationships between each environmental variable and the local contribution to β-diversity (LCBD) of plant communities using linear and quadratic regression models. Model selection was based on Akaike’s Information Criterion (AIC). If the AIC difference between two models exceeded 10, we selected the model with the lower AIC. For example, when the AIC of the linear model was at least 10 units lower than the quadratic model, we interpreted the relationship as best described by a linear trend. Given the potential role of rare species, we examined their effect on community-level LCBD. Rare species were defined as those occurring in less than 5% of the sampling plots. The relative abundance of rare species within each community was computed, and we investigated its correlation with species richness.

To further evaluate the influence of environmental variables on LCBD, we employed stepwise regression and variance partitioning. Given the potential confounding effects of multicollinearity, we applied VIF thresholds (>10) to filter out redundant variables before implementing multivariate regression models (Supplementary Figure S1). The “step” function was then applied to select variables based on AIC, retaining the model with the lowest AIC. Only statistically significant variables were included in the final model, which represented the optimal set of explanatory predictors.

We constructed a structural equation model (SEM) using the piecewiseSEM package in R to investigate the direct and indirect effects of soil properties, climatic factors, and community characteristics on LCBD (local contribution to β-diversity). Prior to SEM, stepwise regression was conducted via the stats package (base R) to screen variables, whose results informed the initial model structure. Data cleaning and organization were handled by the tidyverse suite (with dplyr as a core module) in R. Model fit was evaluated using AIC, chi-squared tests, and Fisher’s C statistic, while standardized path coefficients quantified variable relationships. The initial model included all assumed paths as well as all plant, climate, and soil data. Non-significant paths (*p* ≥ 0.05) were sequentially removed, leaving only statistically significant links. The final model included the following data: PS, TS, AP, TSN, pH, TN, SR, PCC, and LCBD. All analyses (AIC calculation, stepwise regression, variance partitioning, SEM) were executed in R version 4.4.0, and final figures were produced with Origin 2021.

## 3. Results

### 3.1. Geographic Patterns of Plant Community Ecological Uniqueness

**Figure 2:**
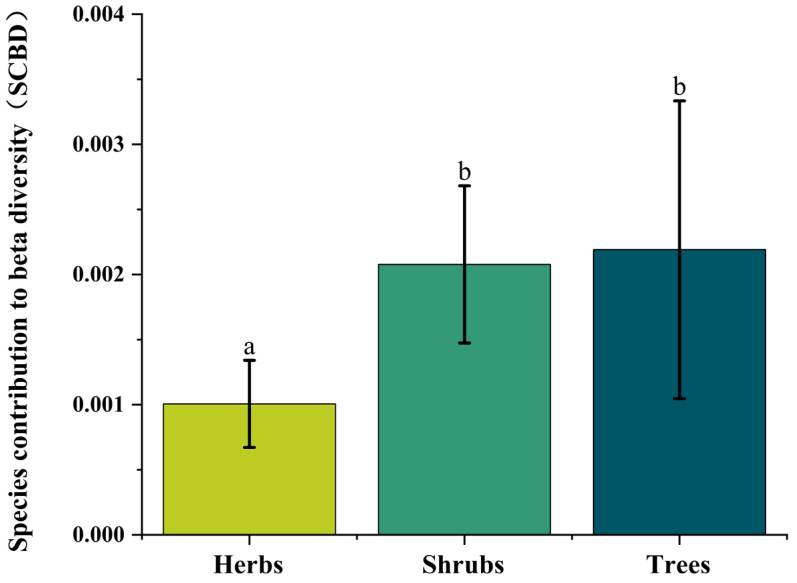
Variation in species contribution to β-diversity (SCBD) across different life forms, presented as mean ± standard error (SE). Different letters denote statistically significant differences among life forms (*p* < 0.05).

The β-diversity of the study area was 0.971. To identify species contributing to community differentiation, we calculated the species contribution to the β-diversity (SCBD). The ten species with the highest SCBD values were *Kobresia pygmaea*, *Potentilla fruticosa*, *Salix oritrepha*, *Caragana versicolor*, *Kobresia vidua*, *Sophora moorcroftiana*, *Potentilla parvifolia*, *Sibiraea angustata*, *Kobresia humilis*, and *Leontopodium pusillum*. These species exhibited SCBD values ranging from 0.012 to 0.228 (Supplementary Table S2). Shrubs and trees contributed more to β-diversity than herbs, with similar magnitudes of contribution between the two woody groups (Figure 2). Local contributions to β-diversity (LCBD) varied from 0.000 to 0.008 across plots, indicating differences in species composition among sampling units.

**Figure 3:**
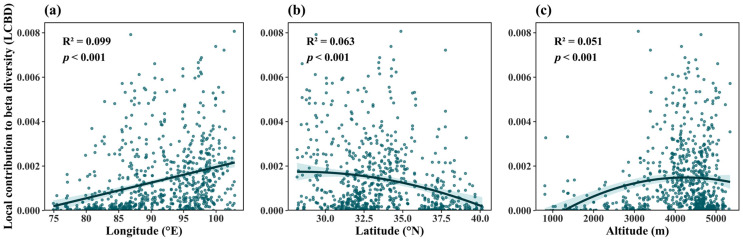
Patterns of plant ecological uniqueness in plant communities across Qinghai-Tibetan Plateau, examined along longitude (**a**), latitude (**b**), and altitude (**c**).

To examine the spatial distribution of ecological uniqueness, we performed univariate linear regressions of LCBD against geographic variables. The analysis revealed a significant positive correlation between LCBD and longitude (Figure 3a, *p* < 0.001), a significant negative correlation with latitude (Figure 3b, *p* < 0.001), and a non-linear relationship with altitude, where LCBD increased initially and then decreased (Figure 3c, *p* < 0.001).

Concurrently, the proportion of rare species exhibited significant positive correlations with both ecological uniqueness (LCBD; Supplementary Figure S1) and geographic gradients (longitude, latitude, elevation; Supplementary Figure S2).

### 3.2. Relationships Between Ecological Uniqueness of Plant Communities and Influencing Factors

**Figure 4:**
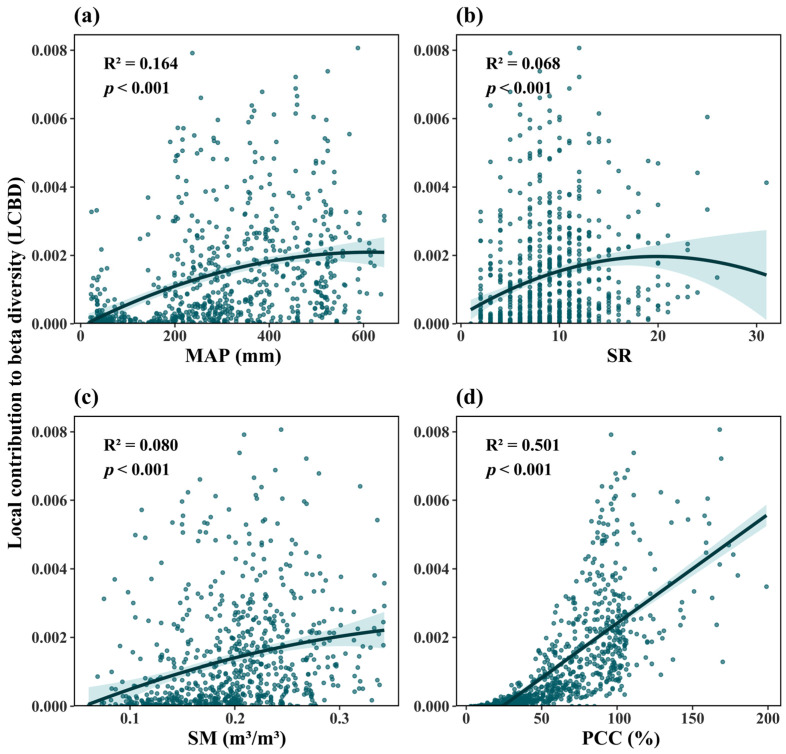
Trends of ecological uniqueness of plant communities across the Qinghai-Tibetan Plateau along mean annual precipitation (MAP) (**a**), species richness (SR) (**b**), soil moisture (SM) (**c**), and plant community coverage (PCC) (**d**).

We employed AIC-based univariate regressions (linear/quadratic) to identify environmental correlates of ecological uniqueness (LCBD). Significant quadratic relationships emerged with species richness, soil moisture, nitrogen content, pH, annual precipitation, precipitation seasonality, and solar radiation (all *p* < 0.001), while plant community coverage showed a linear relationship (*p* < 0.001; Figure 4, Table 1).

**Figure 5:**
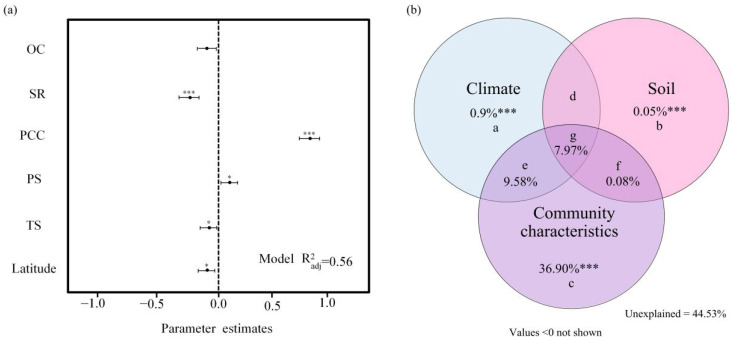
Relative effects of different variables on the ecological uniqueness of plant communities. (**a**) Parameter estimates and their corresponding 95% confidence intervals for each variable included in the final models. (**b**) The influence of climate (MAT, TS, MAP, PS, SRAD), soil (SM, TN, TP, pH, BD, OC), and community characteristics(SR and PCC) on the ecological uniqueness of plant communities. *** *p* < 0.001.* *p* < 0.05.

Stepwise regression refined these drivers, revealing five key predictors: species richness, temperature seasonality, community coverage, precipitation seasonality, and soil organic carbon (adj. R^2^ = 0.56; Figure 5a). Community coverage exerted the strongest influence on LCBD variation, though all factors contributed significantly (*p* < 0.001).

In addition, we performed variance partitioning to identify the main drivers of LCBD. The results indicated that community characteristics, climatic factors, and soil variables together explained 55.47% of LCBD’s spatial variation. Specifically, community characteristics accounted for 36.90%, climate for 0.90%, and soil variables for 0.05% of the variation (Figure 5b). Each factor’s individual contribution was statistically significant (*p* < 0.001). Interactions among variables contributed an additional 7.97%, with climate and community characteristics jointly explaining 9.58% and soil and community characteristics contributing 0.08%.

**Figure 6:**
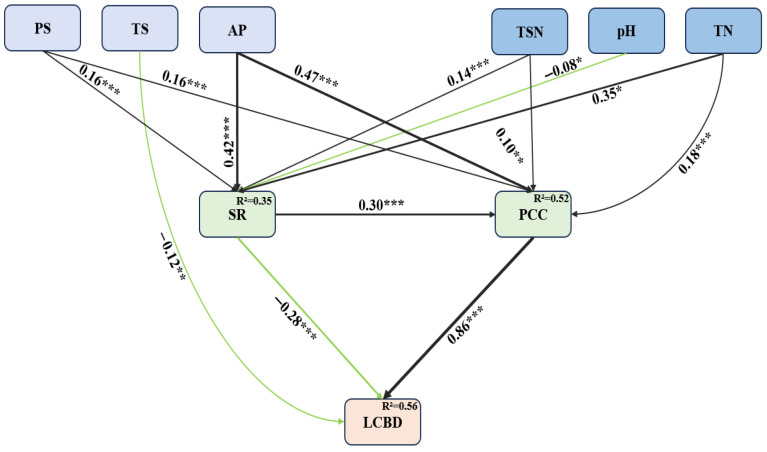
Structural equation models (SEM) illustrating the influences of climatic (PS, TS, and AP), edaphic variables (TSN, pH, AND TN), and community characteristics (species richness and vegetation coverage of community) on the ecological uniqueness of the community (LCBD). Arrows indicate the directional relationships between variables, with black and green lines represent positive and negative influences, respectively. Standardized path-coefficients are presented for statistically significant paths (*** *p* < 0.001, ** *p* < 0.01, * *p* < 0.05), and the width of each path corresponds to the magnitude of its coefficient. The R^2^ values are provided with the response variable.

Structural equation modelling (SEM) supported a good model fit (AIC = −4279.033, Fisher’s C = 19.12, df = 6, *p* = 0.184, Figure 6). Standardized path coefficients varied significantly across space. Soil variables had no direct effect on LCBD, while temperature seasonality showed a significant direct negative effect. In contrast, precipitation seasonality, mean annual precipitation, soil moisture, soil pH, and nitrogen content influenced LCBD indirectly through species richness and community coverage. A significant positive relationship was found between species richness and plant coverage, suggesting that increases in species richness may promote higher community coverage.

## 4. Discussion

### 4.1. Spatial Patterns of Ecological Uniqueness in Plant Communities

Our findings resonate with several key concepts raised in the Introduction. First, aligning with our first hypothesis, LCBD exhibited significant longitudinal increases, latitudinal decreases, and unimodal elevational trends (Figure 3), driven by topographic heterogeneity and rare species distribution. This spatial pattern underscores the role of geographic gradients in shaping ecological uniqueness. Second, consistent with Legendre and De Cáceres [17], LCBD and SCBD effectively captured community-level and species-level uniqueness across the Qinghai-Tibetan Plateau, affirming their utility in terrestrial ecosystems—a critical advance beyond their predominant application in freshwater studies [19,26,27,28,29].

The spatial distribution of species richness and its underlying mechanisms have long been a central focus in both biology and geography [38,39]. Numerous studies have demonstrated that most taxonomic groups exhibit a latitudinal gradient in species richness, typically declining from the equator toward the poles [40,41]. In our study system, local contributions to β-diversity (LCBD) decreased with increasing latitude [42,43], aligning with the negative correlation predicted by Rapport’s rule in specific contexts [44]. However, broader syntheses reveal inconsistent latitudinal patterns in β-diversity across studies—including positive, negative, or non-significant relationships [45,46]. These inconsistencies likely arise from variations in analytical methods, taxonomic groups, spatial scales, and regional context. Along the longitudinal gradient, LCBD increased with longitude. With elevation, LCBD showed a unimodal pattern—initially increasing, then declining at higher altitudes.

The Qinghai-Tibetan Plateau, the highest in the world, exhibits a distinct alpine plateau climate. Its complex topography and surrounding mountain ranges drive strong spatial variations in hydrothermal conditions (Supplementary Table S3). Site-specific analysis showed that precipitation seasonality increased and then decreased with rising longitude and elevation and exhibited a reverse trend with latitude—first decreasing, then increasing (with eastward longitude and northward latitude considered increasing). These findings are consistent with previous studies on precipitation variability across the Qinghai-Tibetan Plateau [46,47].

Previous studies have suggested that the proportion of rare species in a community significantly influences LCBD [24,48]. Our findings support this relationship: the proportion of rare species was strongly correlated with LCBD in plant communities (Supplementary Figure S1). In addition, rare species proportion exhibited significant correlations with longitude, latitude, and elevation (*p* < 0.001) (Supplementary Figure S2). Both LCBD and the proportion of rare species displayed similar spatial patterns. Along longitudinal and latitudinal gradients, LCBD first decreased and then increased. With elevation, both metrics initially increased and then decreased. These trends suggest that spatial heterogeneity in ecological factors—particularly water availability and the distribution of rare species—driven by variations in topography contributes to the uneven spatial patterns of plant community uniqueness on the Qinghai-Tibetan Plateau.

In our study plots, the species contribution β-diversity (SCBD) was significantly higher for shrubs and trees than for herbaceous species. Similar patterns were reported by Wang et al. (2023) in temperate desert plant communities in China [49]. Their study area substantially overlapped with ours and shared similar environmental conditions. However, Wang et al. (2022) found contrasting results in the Ebinur Lake Basin, where herbaceous species exhibited significantly higher SCBD than shrubs and trees [50]. Here, woody plants (shrubs/small trees) maintained stable relative abundance due to conservative ecological strategies. In contrast, herbaceous species exhibited higher dominance variability (e.g., cover shifts across gradients), driving their greater contribution to β-diversity (reflected in elevated SCBD values). In temperate deserts across China, shrubs and subshrubs commonly dominate, thought dominant species compositions vary across regions. Due to the limited presence of herbaceous plants in these communities, their overall contribution to β-diversity remains relatively low [48,50]. Other studies have identified strong associations between SCBD and species richness, as well as leaf thickness. Functional trait differences among trees, shrubs, and herbs are substantial, reflecting their distinct genetic backgrounds. These traits are shaped by environmental adaptation. Recent studies further corroborate that variation in plant functional traits across arid regions is primarily regulated by environmental gradients, particularly precipitation [51], while spatial patterns of carbon stocks in these regions are determined by interactive effects between ecosystem types and soil factors [52], offering a multidimensional perspective for understanding ecological processes in drylands. While species identity and richness typically align with environmental conditions, total species richness may also be influenced by environmental changes or anthropogenic disturbances. Inconsistencies in SCBD patterns across studies likely result from variations in habitat conditions, dominant species composition, and community structure—factors shaped by local environmental contexts.

### 4.2. Drivers of Ecological Uniqueness Patterns in Plant Communities

Environmental factors—including soil moisture, topography, and climate—demonstrate significant yet context-dependent influences on community ecological uniqueness [42,43]. Their relative effects vary substantially across ecosystems and community types. For instance, Wang et al. (2016) observed co-regulation of LCBD by species richness and soil moisture in desert communities, with factor importance shifting according to species composition [50]. Similarly, Yao et al. (2021) reported differential environmental controls across forest types: soil nutrients significantly influenced ecological uniqueness in subtropical mixed forests but not in temperate coniferous forests [24]. This ecosystem-specific pattern extends to aquatic environments, where Vilmi et al. (2017) found regional environmental drivers predominated in stream diatom communities, whereas spatial factors dominated in lake systems [19]. In our study, soil, climate, and community characteristics each had significant independent effects on the LCBD, together explaining 55.47% of the spatial variation. Among these factors, community characteristics contributed the most (36.90%, *p* < 0.001), far surpassing the influence of climate (0.9%, *p* < 0.001) and soil (0.05%, *p* < 0.001). These results highlight the dominant role of intrinsic community traits in shaping species composition, consistent with previous findings [24,25,27]. These results provide novel insights into the drivers of ecological uniqueness in high-altitude plant communities. Specifically, our plateau-scale analysis reveals that community characteristics (e.g., species richness, coverage) play a more significant role than climatic and edaphic factors in regulating uniqueness. This pattern contrasts with findings from many lowland ecosystems [24,31], highlighting the unique assembly processes occurring on the Qinghai-Tibetan Plateau.

Soil and climate are widely recognized as key drivers of plant community β-diversity [24,53,54]. Structural equation modeling in this study further revealed that both factors significantly influenced community characteristics, which in turn affected LCBD. Specifically, climate and soil explained 35% and 52% of the variation in species richness and community coverage, respectively. Critically, our SEM identified a significant positive relationship between species richness and vegetation coverage, indicating that higher species richness promotes increased ground cover through complementary resource use and niche filling. These findings align with those of Fu et al. (2025), who examined herbaceous plant diversity in plateau climate zones [55]. They also reinforce prior evidence that soil properties affect species richness [54,56] and that climate influences plant diversity patterns [57,58]. Our findings support a “dual-engine” model of community assembly on the Qinghai-Tibetan Plateau, driven by the interactive effects of soil and climate. Soil nutrient enrichment and pH suppression exert opposing but balanced effects, while climatic variables—particularly precipitation—enhance the positive influence of soil nutrients. This interaction accounts for 35–52% of the variation in community characteristics and indirectly shapes community β-diversity by altering species richness and plant coverage. These insights align with the “water–heat–nutrient” co-regulation framework for alpine ecosystems and help quantify the pathways through which environmental factors interact. Future research should incorporate long-term monitoring to clarify the roles of freeze–thaw cycles, microbial processes, and anthropogenic disturbances in modulating soil–climate interactions.

### 4.3. Non-Linear Relationship Between Ecological Uniqueness and Species Richness

The present study revealed that, in addition to climatic and edaphic factors, species richness and plant community coverage significantly affect LCBD. These findings are consistent with previous studies [24,43,49]. However, the relationship between species richness and ecological uniqueness remains inconsistent across studies [25,26]. Our analysis revealed a non-linear, hump-shaped (quadratic) correlation between species richness and ecological uniqueness, with the highest point observed at around 20 species. Similarly, a quadratic relationship emerged between the proportion of rare species and ecological uniqueness, with uniqueness peaking when rare species composed approximately 50–55% of the community. Additionally, the proportion of rare species followed an inverse U-shaped pattern with species richness, peaking at 10–15 species (Supplementary Figure S3). As richness surpassed 20 species, the proportion of rare species increased while ecological uniqueness declined (Supplementary Figure S1). These patterns suggest a threshold effect: at moderate species richness, niche complementarity enhances resource-use efficiency and maximizes ecological uniqueness. Beyond this threshold, intensified competition fosters functional redundancy, reducing community uniqueness. This interpretation aligns with theoretical work by Hernández-García et al. (2008), who showed that when interspecific competition kernels are positive definite (e.g., Gaussian), niche complementarity promotes stable coexistence and efficient resource use [59]. In contrast, non-positive definite kernels or parameter imbalances (e.g., box-shaped kernels) lead to species clustering and functional redundancy, consistent with our high-diversity findings. Although previous studies have examined links between functional diversity and ecological uniqueness [48], our analysis did not incorporate additional plant functional traits. Future research should explore how other trait axes of alpine flora—particularly on the Qinghai-Tibetan Plateau—influence ecological uniqueness and underlying mechanisms, thereby advancing understanding of biodiversity–function relationships in high-altitude ecosystems.

Despite comprehensive analyses, several limitations warrant attention. First, plot size adjustments necessitated by topographic constraints may introduce comparability issues in community coverage estimation, particularly for rare species detection. Furthermore, the non-uniform spatial distribution of sampling points resulted in unsampled areas and spatial gaps, potentially biasing β-diversity estimation. Future efforts should prioritize increased sampling intensity, particularly in undersampled regions, to enhance the representativeness and accuracy of the findings. Third, the timeliness of the dataset warrants consideration. While the fieldwork (2018–2021) captured seasonal and inter-annual variations [60,61,62], and the observed ecological patterns (e.g., spatial relationships of ecological uniqueness driven by species richness, community coverage, and environmental gradients [63,64,65]) are expected to remain relevant due to the inherent stability of high-altitude ecosystems on the Qinghai-Tibetan Plateau and the relatively slow rates of change in its remote, low-disturbance areas [65,66,67], the dynamic nature of ecosystems necessitates ongoing monitoring. Incorporating longitudinal surveys and updated datasets in future studies is crucial to more comprehensively capture temporal dynamics, assess the persistence of observed patterns, and detect any potential gradual shifts driven by factors like long-term climate change. To address this, we plan to conduct follow-up field surveys in the coming years [68,69,70] to evaluate potential changes in community composition and ecological uniqueness.

## 5. Conclusions

This study systematically analyzed spatial patterns and mechanisms of ecological uniqueness in Qinghai-Tibetan Plateau plant communities. Key findings: (1) Ecological uniqueness exhibits a clear geographical gradient shaped by longitude, latitude, and elevation, with rare species as key mediators—aligning with the ‘water-heat-nutrient co-regulation’ theory; (2) Species richness and plant coverage exert the strongest direct influence, highlighting local assembly’s centrality; (3) Climate–soil synergies indirectly affect uniqueness via coupled water–nutrient dynamics, driving functional trait variation. We provide the first quantification of path coefficients for these interactions. Conservation priorities should target regions exhibiting high ecological uniqueness (LCBD) to preserve their distinctive species assemblages and associated ecological functions. This imperative arises because our findings demonstrate that high-LCBD areas harbor a significantly higher proportion of rare species (Supplementary Figure S1), which are often locally endemic or specialized, and possess unique species compositions, contributing disproportionately to overall β-diversity. Furthermore, these unique assemblages, frequently characterized by a greater influence of woody species (shrubs and trees) on β-diversity (Figure 2), emerge from specific environmental contexts shaped by the interplay of soil, climate, and local community characteristics like species richness and coverage (Figure 5 and Figure 6). Protecting these ecologically unique sites is therefore crucial for maintaining the compositional heterogeneity (β-diversity) of the Qinghai-Tibetan Plateau’s plant communities and safeguarding the irreplaceable ecological functions supported by these distinct species combinations.

## Figures and Tables

**Table 1:** Relationship between the ecological uniqueness of plant communities and single environmental variable.

Variable	R^2^	Slope	*p*
Species richness (SR)	0.068	UM	<0.001
Soil moisture (SM)	0.080	DU	<0.001
Plant community coverage (PCC)	0.501	0.0006/L+	<0.001
Soil total nitrogen content (TSN)	0.014	UD	<0.001
Soil pH (pH)	0.015	DU	<0.001
Mean annual precipitation (MAP)	0.164	DU	<0.001
Precipitation seasonality (PS)	0.046	DU	<0.001
Solar radiation (SRAD)	0.0215	DD	<0.001

UM indicates a quadratic pattern, where the values first increase and then decrease. DU represents an upward-opening quadratic pattern indicating a gradual increase. UD represents an upward-opening quadratic pattern indicating a gradual decrease. DD indicates a quadratic pattern characterized by a gradual decrease. L+ Significant Positive Linear.

## Data Availability

The original data presented in this study are openly available in the Chinese Journal of Plant Ecology repository at https://www.plant-ecology.com/CN/10.17521/cjpe.2022.0174, DOI: 10.17521/cjpe.2022.0174, accessed on 11 September 2023.

## Supplementary Materials

The following supporting information can be downloaded at: https://www.mdpi.com/article/10.3390/plants14152379/s1, Figure S1: Plant community ecological specificity in relation to the proportion of rare species to the total number of species in the community and relative abundance (relative cover); Figure S2: Patterns of change in the proportion of rare species to the total number of species in the community along longitudinal, latitudinal and altitudinal gradients; Figure S3: Patterns of change in the proportion of rare species to the total number of species in the community and in the relative cover of rare species as a function of the gradient of species richness in the community; Table S1: Correlations between environmental variables; Table S2: Contribution of important plant species to beta diversity on the Tibetan Plateau; Table S3: Correlation between environmental factors and geographic factors on the Tibetan Plateau.
