# Supplementary material for: Patterns and Determinants of Ecological Uniqueness in Plant Communities on the Qinghai-Tibetan Plateau
Source: Plants (Basel). 2025 Aug 1;14(15):2379. doi: 10.3390/plants14152379 (PMC12349686; doi:10.3390/plants14152379)

Supplementary Table S1

Correlations between environmental variables

Related Analysis

	TS	PS	AP	MAT	TSN	SRAD	SOM	PH	TN	SR	PCC	TP
TS	1											
PS	-.317**	1										
AP	-.685**	0.036	1									
MAT	.449**	-.150**	-.323**	1								
TSN	-.293**	0.045	.585**	-.180**	1							
SRAD	.478**	-.354**	-.216**	.203**	-.279**	1						
SOM	-.286**	0.05	.502**	-.227**	.389**	-0.066	1					
PH	-.111**	.286**	.111**	-.133**	.267**	-.174**	.146**	1				
TN	-.348**	.076*	.560**	-.286**	.424**	-.080*	.975**	.137**	1			
SR	-.325**	.119**	.428**	-.186**	.310**	-.105**	.390**	0.046	.420**	1		
PCC	-.255**	.213**	.328**	-.073*	.241**	-.138**	.299**	.089*	.310**	0.062	1	
TP	-.362**	.171**	.310**	-.216**	.208**	-.156**	.482**	.085*	.498**	.250**	.124**	1

Supplementary Table S2

Contribution of important plant species to beta diversity on the Tibetan Plateau

Scientific name	Latin of family	Latin of genera	SCBD	Frequency (%)
Kobresia pygmaea	Cyperaceae	Kobresia	0.228235006	23.21899736
Potentilla fruticosa	Rosaceae	Potentilla	0.050482006	5.80474934
Salix oritrepha	Salicaceae	Salix	0.033574313	2.506596306
Caragana versicolor	Fabaceae	Caragana	0.032091916	2.110817942
Kobresia vidua	Cyperaceae	Kobresia	0.027106876	2.110817942
Sophora moorcroftiana	Fabaceae	Sophora	0.021596838	1.319261214
Potentilla parvifolia	Rosaceae	Potentilla	0.018783709	6.860158311
Sibiraea angustata	Rosaceae	Sibiraea	0.01472904	1.846965699
Kobresia humilis	Cyperaceae	Kobresia	0.01216149	5.145118734
Leontopodium pusillum	Asteraceae	Leontopodium	0.01216008	14.24802111
Polygonum viviparum	Polygonaceae	Polygonum	0.011794082	5.80474934
Potentilla bifurca	Rosaceae	Potentilla	0.011519817	15.56728232
Stipa purpurea	Poaceae	Stipa	0.010015078	15.56728232
Aster flaccidus	Asteraceae	Aster	0.009944237	6.200527704
Poaceae	Poaceae		0.009799678	6.068601583
Potentilla saundersiana	Rosaceae	Potentilla	0.00942236	19.78891821
Lancea tibetica	Mazaceae	Lancea	0.009292691	6.72823219
Potentilla anserina	Rosaceae	Potentilla	0.009001697	2.90237467
Phragmites australis	Poaceae	Phragmites	0.008962893	3.166226913
Kobresia royleana	Cyperaceae	Kobresia	0.008728507	4.089709763
Poa albertii	Poaceae	Poa	0.008722121	3.298153034
Kobresia tibetica	Cyperaceae	Kobresia	0.008607454	2.770448549
Picea likiangensis var. rubescens	Pinaceae	Picea	0.008211647	0.263852243
Stipa roborowskyi	Poaceae	Stipa	0.007917749	6.860158311
Ajuga lupulina	Lamiaceae	Ajuga	0.007460907	1.715039578
Potentilla bifurca var. humilior	Rosaceae	Potentilla	0.00746066	8.179419525
Tamarix hohenackeri	Tamaricaceae	Tamarix	0.006933883	1.978891821
Juniperus squamata	Cupressaceae	Juniperus	0.006750556	0.395778364
Potentilla plumosa	Rosaceae	Potentilla	0.006413163	2.110817942
Lonicera rupicola var. minuta	Caprifoliaceae	Lonicera	0.005993495	1.319261214
Astragalus polycladus	Fabaceae	Astragalus	0.005782966	7.255936675
Carex moorcroftii	Cyperaceae	Carex	0.005774119	4.089709763
Saussurea arenaria	Asteraceae	Saussurea	0.005641877	3.430079156
Kobresia	Cyperaceae	Kobresia	0.005582058	1.583113456
Ligularia virgaurea	Asteraceae	Ligularia	0.005263068	2.374670185
Artemisia wellbyi	Asteraceae	Artemisia	0.005202307	6.332453826

Achnatherum duthiei	Poaceae	Achnatherum	0.005182967	6.332453826
Lasiocaryum densiflorum	Boraginaceae	Lasiocaryum	0.005129038	12.00527704
Kobresia capillifolia	Cyperaceae	Kobresia	0.004732528	1.715039578
Juniperus convallium	Cupressaceae	Juniperus	0.004219317	0.263852243

Supplementary Table S3

Correlation between environmental factors and geographic factors on the Tibetan Plateau

Related Analysis

	longtitude	latitude	elevation
TS	-0.022	0.896**	-0.539**
PS	0.047	-0.359**	0.255**
AP	0.613**	-0.354**	0.127**
MAT	-0.026	0.081*	-0.675**
TSN	0.506**	-0.079*	-0.054
SRAD	0.015	0.517**	-0.437**
SOM	0.564**	-0.237**	0.136**
PH	0.094**	-0.113**	0.130**
TN	0.526**	-0.275**	0.198**
SR	0.420**	-0.252**	0.153**
PCC	0.349**	-0.170**	0.035
LCBD	0.430**	-0.252**	0.126**
TP	0.094**	-0.385**	0.272**

**, P < 0.001.

*, P < 0.005.

Supplementary Figure S1

Plant community ecological specificity in relation to the proportion of rare species to the total number of species in the community and relative abundance (relative cover)

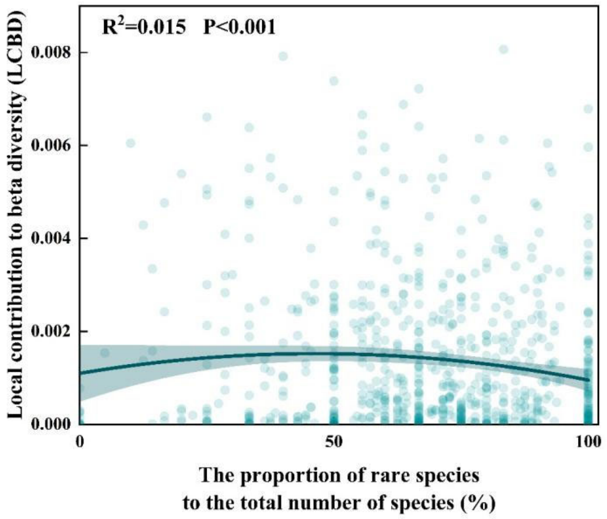

Supplementary Figure S2

Patterns of change in the proportion of rare species to the total number of species in the community along longitudinal, latitudinal and altitudinal gradients

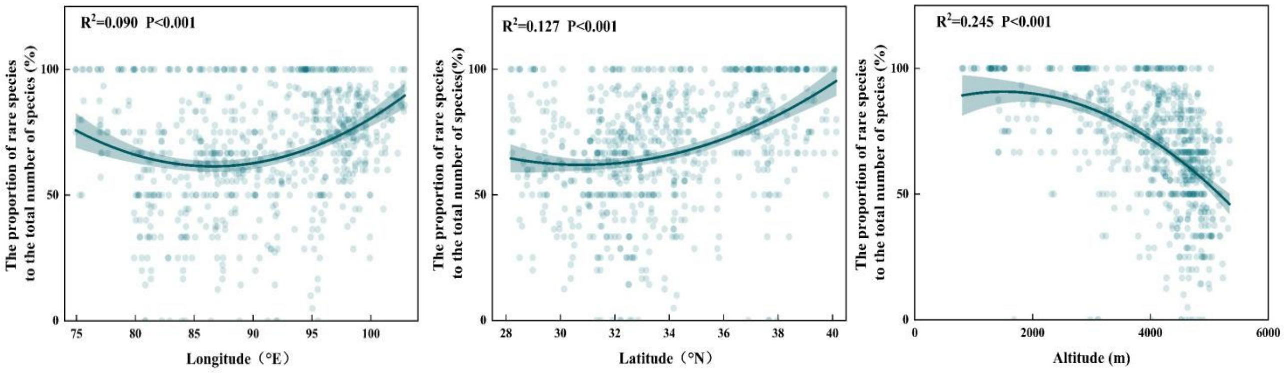

Supplementary Figure S3

Patterns of change in the proportion of rare species to the total number of species in the community and in the relative cover of rare species as a function of the gradient of species richness in the community

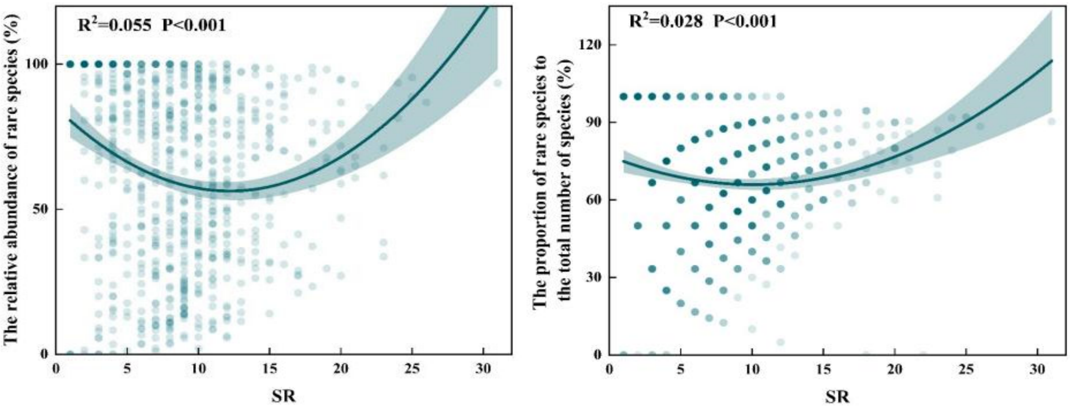

Supplement: Supplementary file 1 [file plants-14-02379-s001.zip › plants-3703423-supplementary.pdf]
